# The effect of sexual behavior on HIV-1 seroconversion is mediated by the gut microbiome and proinflammatory cytokines

**DOI:** 10.21203/rs.3.rs-3868545/v1

**Published:** 2024-01-24

**Authors:** Huang Lin, Yue Chen, Grace Abror, Meaghan Price, Alison Morris, Jing Sun, Frank Palella, Kara W. Chew, Todd T. Brown, Charles R. Rinaldo, Shyamal D. Peddada

**Affiliations:** 1 Co-first authors.; 2 Co-senior authors.; 3 Biostatistics and Computational Biology, National Institute of Environmental Health Sciences (NIH), Research Triangle Park, NC USA.; 4 Division of Infectious Diseases, Department of Medicine, University of Pittsburgh, Pittsburgh, PA USA.; 5 Department of Infectious Diseases and Microbiology, School of Public Health, University of Pittsburgh, Pittsburgh, PA USA.; 6 Division of Pulmonary, Allergy and Critical Care Medicine, Department of Medicine, University of Pittsburgh School of Medicine, Pittsburgh, PA USA; 7 Department of Epidemiology, The Johns Hopkins Bloomberg School of Public Health, Baltimore, MD USA.; 8 Department of Medicine, Feinberg School of Medicine, Northwestern University, Chicago, IL USA; 9 School of Medicine, University of California at Los Angeles, Los Angeles, CA, USA

## Abstract

The association between HIV-1 seroconversion and gut dysbiosis is well documented, and its association with sexual activity is also widely recognized. However, it is not known whether the gut dysbiosis mediates the effects of high-risk sexual behavior on HIV-1 seroconversion. In this report we focused on men who engaged in high-risk sexual behavior where they had receptive anal intercourse with multiple men. We demonstrate that proinflammatory cytokines, sCD14 and sCD163, and gut microbiota mediate the effects of this high-risk sexual behavior on subsequent HIV seroconversion. We discovered changes in the gut microbial ecology, prior to seroconversion, both in terms of the composition as well as inter-relationships among the commensal species. Furthermore, these changes correlate with future HIV seroconversion. Specifically, as the number of sexual partners increased, we discovered in a “dose-response” manner, a decrease in the abundance of commensal and short-chain fatty acid-producing species, *A. muciniphila, B. caccae, B. fragilis, B. uniformis, Bacteroides spp., Butyricimonas spp*., and *Odoribacter spp,* and an increase in proinflammatory species *Dehalobacterium spp*. and *Methanobrevibacter spp*. These changes were also observed among subsequent HIV seroconverters. Interestingly, we also discovered a reduction in correlations among these commensal and short-chain fatty acid producing bacteria in a “dose-response” manner with the number of sexual partners. Our mediation analysis not only provides a conceptual model for the disease process but also provides clues for future clinical interventions that will manipulate the gut microbiota to treat high-risk subjects to prevent HIV seroconversion.

## Introduction

Since the beginning of the HIV-1 epidemic in the USA, sexual contact among men who have sex with men (MSM) has been the primary route of HIV-1 transmission in the US^1^. Sexual transmission currently accounts for more than 90% of new HIV-1 infections in the US, with 68% transmitted through sexual contact between MSM^2,3^. Anal intercourse is the riskiest sexual practice for acquiring or transmitting HIV-1, with the receptive partner at higher risk than the insertive partner^4–8^. Advances in our understanding of HIV-1 pathogenesis have revealed the critical role of cytokines, the gut microbiome, and the microbiome byproduct short-chain fatty acids (SCFAs) in HIV-1 transmission and disease progression^9^. Gut microbiome dysbiosis can increase local inflammatory cytokine production, thereby activating CD4^+^ T cells and increasing HIV-1 co-receptor CCR5 expression on CD4^+^ T cells in gut-associated lymphoid tissues (GALT), the cells preferentially infected by HIV-1^10–12^. The presence of activated CD4^+^ T cells in the rectum and anus makes MSM who practice unprotected anal receptive sexual intercourse particularly susceptible to HIV-1 infection. Moreover, inflammation and immune activation of GALT lead to the translocation of gut bacteria and bacterial products into the blood circulation, resulting in systemic immune activation that fuels the dissemination of HIV-1 when initial viral exposure occurs in local GALT^4,13^. Cytokines, as key mediators of immune responses, can modulate the activation and function of immune cells, including CD4^+^ T cells that are the primary target of HIV-1. Elevated systemic levels of pro-inflammatory cytokines contribute to a heightened state of immune activation, rendering individuals more susceptible to HIV-1 infection^14–16^. SCFAs produced by the gut microbiota through the fermentation of dietary fiber also possess immunomodulatory and anti-inflammatory properties^17^. SCFAs can influence the gut barrier function and modulate the local immune response, potentially affecting HIV-1 transmission and dissemination^18^.

Compared to men who have sex with women (MSW), MSM – regardless of HIV-1 serostatus – have a Prevotella-rich fecal^19,20^ and rectal mucosal^5^ microbiome, as well as a rectal mucosal immune activation profile. We previously reported that high levels of plasma inflammatory cytokines and gut microbiome profiles characterized by increased Prevotella and decreased Bacteroides a few months before HIV-1 infection were significantly associated with subsequent, primary HIV-1 infection in over 100 MSM from the 1984–85 Multicenter AIDS Cohort Study (MACS) cohort^21^. Consistent with this finding, the odds of seroconversion were highly positively correlated with the ratio of *Prevotellaceae* to *Bacteroideceae*. Altered microbiome in MSM prior to HIV-1 seroconversion was confirmed by Fulcher, et al.^22^ in a study of 2014–2018 non-MACS HIV-1 research cohorts. However, to the best of our knowledge, the interactions among sexual behavior, biomarker levels including cytokines, the gut microbiome, and SCFA with HIV-1 infection has not been investigated.

Capitalizing on the valuable longitudinal data collected before and after HIV-1 seroconversion in the MACS (Extended Data Fig. 1), in the current study we have comprehensively analyzed: (1) the associations and interactions among self-reported sexual behaviors associated with increased risk for HIV (number of partners with whom a participant had receptive anal intercourse, the primary exposure variable in this study), systemic inflammatory cytokine levels, gut microbiome, and SCFAs before HIV-1 infection in MSM participants with or without subsequent HIV-1 seroconversion (the outcome variable in this study), and (2) whether changes in these biomarker levels can be considered as mediators through which sexual behavior impacts HIV-1 seroconversion. Achieving these objectives is important to elucidate the complex interplay among sexual behavior, gut microbiome, systemic inflammation, and HIV-1 transmission risk.

## Results

### Study participants and data information

We analyzed plasma cytokine levels, SCFA, and microbiome data of the same study participants (N = 241) from our previous investigation^21^, considering the participants’ reported sexual activity. The cohort was exclusively MSM, and the average age (+/− standard deviation [SD]) of study participants was 41 +/− 16 years (Table 1). The majority of these 1984–85 MACS participants were white (95%) and achieved undergraduate or post-graduate college degrees (63%). The group included a higher number of non-smokers, with 57% either having never smoked or former smokers, and current smokers comprising 42% of the total. A higher percentage of participants (56%) were categorized as heavy drinkers (consumption of three or more drinks per day at least once a month), as opposed to low drinkers (42%, defined as consumption of less than two drinks per day no more than once a month). Prevalent oral bacterial antibiotic use was reported by 53% of the cohort. Approximately 16% of participants reported having had at least one sexually transmitted infection (STI), including syphilis, non-specific/nongonococcal urethritis, chlamydia, herpes, and gonorrhea. A notable proportion (81%) reported past use of one or more substances including marijuana, poppers, cocaine, uppers, ecstasy, XTC, X or MDMA, heroin, speedball, PCP, downers, ethyl chloride, GHB, or other unspecified drugs. About 40% of the participants were hepatitis B virus (HBV) negative (anti-HBc negative and HBsAg negative), 55% had resolved HBV infection (anti-HBc positive and HBsAg negative), and 3% were HBV positive at the time of the survey (HBsAg positive). About 95% of participants were hepatitis C virus antibody negative, 2.5% were positive, and the remaining 2.5% had missing test results.

**Figure 1:**
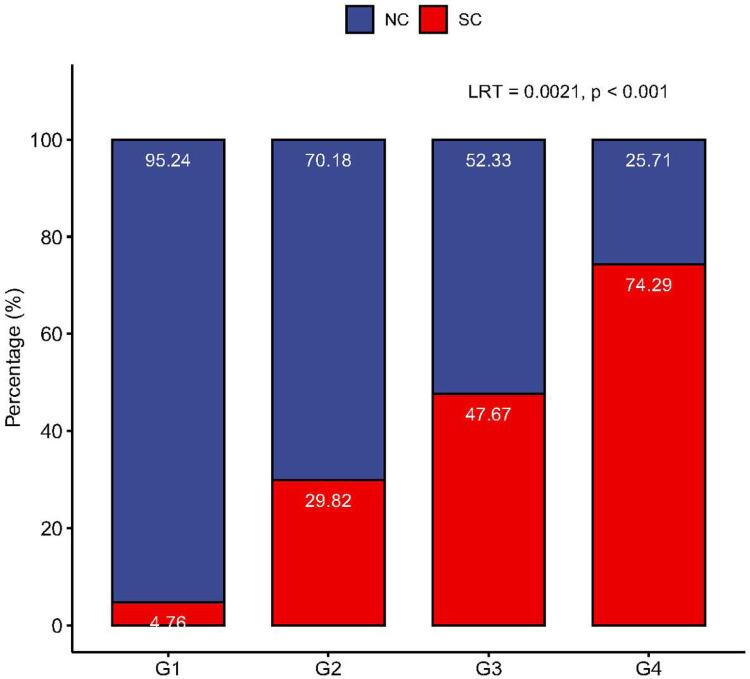
Seroconversion rates demonstrate a monotonic increase in correlation with the number of partners a participant had receptive anal intercourse with. The X-axis categorizes participants into exposure groups, ranging from Group 1 (G1) through Group 4 (G4). The Y-axis represents the percentage distribution of future seroconverters (SC, depicted in red) and negative controls (NC, depicted in blue). Combined, these percentages total 100%. As participants move from G1 to G4, indicative of an increase in the number of partners, there is a marked rise in the percentage of SC. This increase is statistically signicant supported by the likelihood ratio test (LRT) statistics of 0.0021 and a p-value of less than 0.001, as determined by the constrained linear mixed effects (CLME) test, suggesting a monotonic increasing trend.

We found that the seroconverter (SC) rates (relative to seronegative control (NC)) monotonically increased with the number of sexual partners with whom a participant had receptive anal intercourse during the past 2 years (p < 0.001 Fig. 1). This finding suggests that the risk of HIV-1 seroconversion increases with the number of partners a man had receptive anal intercourse with.

**Figure 2:**
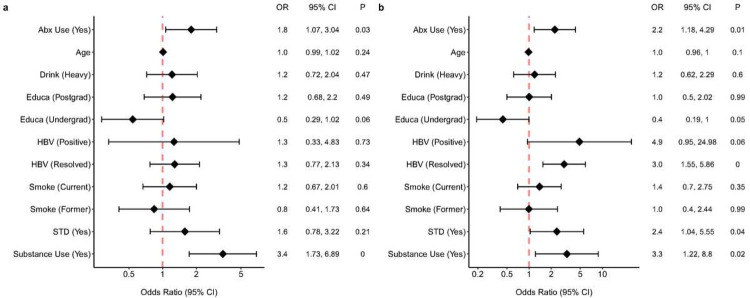
Forest plot depicting the associations of demographic, clinical, and behavior features with (a) sexual exposure groups and (b) the seroconversion status outcome. Results were obtained using (a) ordinal logistic regression models and (b) logistic regression models. The X-axis denotes the odds ratio (OR), while the Y-axis lists the various demographic and clinical characteristics. Each feature’s effect size (OR) is symbolized by a diamond. An accompanying horizontal line represents the 95% condence interval (CI), indicating the range in which the actual effect size is likely to reside. A vertical red line at the OR of 1.0 serves as a reference for no effect. For each feature, the exact OR, 95% CI, and the p-value (two-sided) — derived from logistic regression models — are presented in an adjacent table.

There were no statistically significant differences observed among the four sexual exposure groups (see Methods), or between the HIV-1 seroconversion status groups, with respect to mean ages, alcohol consumption status, education level, and smoking status (Fig. 2). However, recreational substance use was positively associated with the number of receptive anal intercourse partners (p < 0.01, Fig. 2a) and the risk of seroconversion (p = 0.02, Fig. 2b). These findings suggest that substance use acts as a potential confounder between the sexual exposure groups and outcome variables. Not surprisingly, antibiotic usage was positively associated with sexual exposure groups (p = 0.03, Fig. 2a) and with seroconversion (p = 0.01, Fig. 2b). We hypothesized that antibiotic usage did not directly impact HIV-1 seroconversion but instead, influenced levels of the biomarkers (cytokines, gut microbiome, and SCFAs). Consequently, instead of treating antibiotic usage as a confounder, we treated it as a covariate in the subsequent analyses between the sexual exposure groups and biomarkers. Both HBV infection and history of STI positively correlated with HIV seroconversion but were not correlated with the number of partners with whom a participant had receptive anal intercourse (Fig. 2). Hence, rather than being confounders in this study, they could serve as exposures in a different causal pathway leading to HIV-1 seroconversion.

### Association analyses

To evaluate whether changes in biomarker levels, namely, cytokines, the gut microbiome, and SCFAs mediate the effects of sexual behavior on HIV-1 seroconversion, we first examined the associations between levels of these biomarkers with both exposure and outcome. Notably, our previously published work^21^ detailed associations between each of these biomarkers and seroconversion status; In this current study, we (a) investigated the association between the biomarkers, namely, cytokines, SCFAs, and microbiome, and the sexual exposure groups, and (b) reanalyzed the association between the microbiome and seroconversion status using ANCOM-BC2^23^, a recently developed methodology that has better performance characteristics

### Sexual exposure groups and plasma inflammatory cytokine levels.

**Figure 3:**
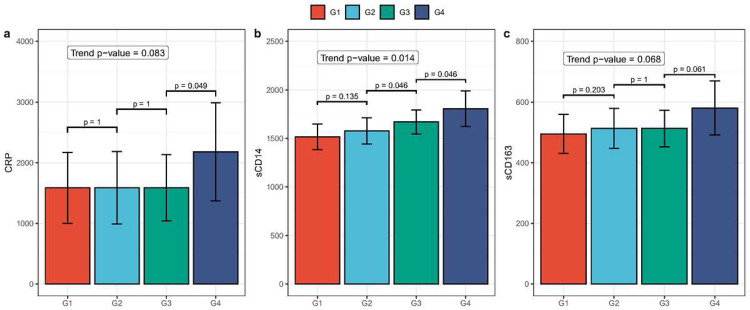
Bar plots demonstrating monotonically increasing trends between exposure groups and plasma levels of (a) CRP, (b) sCD14, and (c) sCD163. The X-axis details the exposure groups, from Group 1 (G1) to Group 4 (G4). The Y-axis reects the cytokine’s effect size, as determined by the constrained linear mixed effects (CLME) model. Each bar’s error bars denote the 95% condence interval (CI). Pairwise p-values (one-sided), used for contrasting the exposure groups, are displayed above the brackets which span the respective bars. The p-value for monotonically increasing trend is provided within the plot.

For each participant, we assessed plasma sCD14, sCD163, IL-6, and LBP to detect monotonic increasing trends with an increase in the number of partners with whom a participant had receptive anal intercourse. As shown in Fig. 3a–c, there is a significant increasing trend in sCD14 levels with sexual exposure groups (p = 0.014), and marginally significant increasing trends in CRP (p = 0.083) and sCD163 (p = 0.068) with sexual activity groups.

### Sexual exposure groups and gut microbiome.

A total of 13,073,544 sequence reads were generated for the 243 stool samples, with the median of 51,125 (range 67 – 126,903) reads per stool sample. The analyses did not demonstrate significant associations between alpha diversity metrics, namely richness and the Shannon diversity index, and the ordered sexual exposure groups. This held true across both increasing trend (Extended Data Fig. 2a-b) and decreasing trend (Extended Data Fig. 2c-d). Furthermore, using the Bray-Curtis dissimilarity, no discernible differences were observed in species Beta diversity across sexual activity groups at the species level (Extended Data Fig. 3).

**Figure 4:**
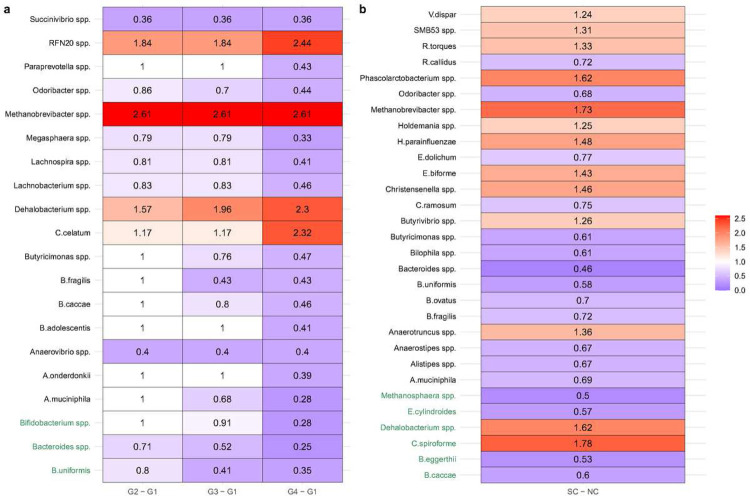
Heatmaps depict the ANCOM-BC2 pattern analysis assessing monotonic increasing or decreasing trends in microbial species abundances concerning (a) exposure groups and (b) outcome. The X-axis delineates contrasts between exposure groups or outcomes, while the Y-axis lists species identied as signicant via ANCOM-BC2 pattern analysis. Species that remained signicant post-adjustment for multiple comparisons using the Benjamini–Yekutieli (BY) are highlighted in green. Fold-changes (natural log base) are superimposed within each cell. The color spectrum, from blue to red with a neutral white midpoint, visualizes the fold-changes. Specically, blue cells indicate reduced abundance relative to the reference group, and red cells signal increased abundance compared to the reference group. A white cell represents no effect, where the fold-change equals 1.

The ANCOM-BC2 pattern analysis was employed to evaluate monotonic increasing or decreasing trends in abundances of microbial species with sexual exposure groups (Fig. 4a). As the number of partners with whom a participant has receptive anal intercourse increased from Group 1 to Group 4, we discovered a significant decreasing trend (p < 0.05) in the abundance of some of the well-known commensal bacteria and those involved in the production of short-chain fatty acids such as *A. muciniphila* (p = 0.001)*, B. uniformis* (p < 0.001)*, Bacteroides spp*. (p < 0.001)*, Bifidobacterium spp*. (p < 0.001)*, A. onderdonkii* (p = 0.007)*, Anaerovibrio spp*. (p = 0.009)*, B. adolescentis* (p = 0.021)*, B. caccae* (p = 0.03)*, B. fragilis* (p = 0.026)*, Butyricimonas spp*. (p = 0.034)*, Lachnobacterium spp*. (p = 0.029)*, Lachnospira spp*. (p = 0.006)*, Megasphaera spp*. (p = 0.006)*, Odoribacter psp*. (p = 0.015)*, Paraprevotella spp*. (p = 0.02), and *Succinivibrio spp*. (p = 0.024). The decreasing trends in *B. uniformis, Bacteroides spp*. and *Bifidobacterium spp*. were significant even after performing multiple testing p-value corrections. On the other hand, there was a significant increasing trend (p < 0.05) in the abundance of *C. celatum* (p = 0.018)*, Dehalobacterium spp*. (p = 0.013)*, Methanobrevibacter spp*. (p = 0.016), and *RFN20 spp*. (p = 0.021) from Group 1 to Group 4 (Fig. 4a).

We implemented an ANCOM-BC2 two-group comparison to discern species exhibiting differential abundance in relation to HIV seroconversion status. Notably, among the species identified as significantly differentially abundant (Fig. 4b), the following species displayed monotonic trends with sexual exposure group: *A. muciniphila* (p = 0.043), *B. caccae* (p = 0.043), *B. fragilis* (p < 0.001), *B. uniformis* (p = 0.006), *Bacteroides spp*. (p < 0.001), *Butyricimonas spp*. (p = 0.001), *Dehalobacterium spp*. (p < 0.001), *Methanobrevibacter spp*. (p = 0.006), and *Odoribacter spp*. (p = 0.013). Interestingly, both *Dehalobacterium spp*. and *Methanobrevibacter spp*. increased in abundance in future SC relative to NC, while the remaining species decreased in abundance among SC relative to NC.

### Sexual exposure groups and fecal short-chain fatty acids (SCFAs).

**Figure 5:**
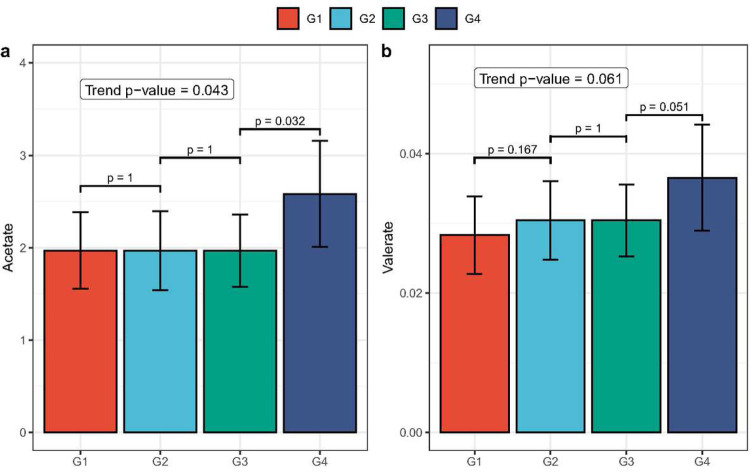
Bar plots demonstrating monotonically increasing trends between exposure groups and short-chain fatty acid (SCFA) levels of (a) acetate and (b) valerate. The X-axis details the exposure groups, from Group 1 (G1) to Group 4 (G4). The Y-axis reects the cytokine’s effect size, as determined by the constrained linear mixed effects (CLME) model. Each bar’s error bars denote the 95\% condence interval (CI). Pairwise p-values (one-sided), used for contrasting the exposure groups, are displayed above the brackets which span the respective bars. The p-value for monotonically increasing trend is provided within the plot.

An increase in the number of partners with whom a participant had receptive anal intercourse was associated with elevated levels of certain SCFAs. Trend test analysis revealed that the increased number of receptive anal intercourse partners was significantly associated with higher levels of acetate (p = 0.043, Fig. 5a). Additionally, a marginally significant association was observed between the increased number of receptive anal intercourse partners and higher levels of valerate (p = 0.061, Fig. 5b). To investigate whether sexual behaviors have lag effects on SCFA levels, we also assessed the trends between groups and SCFAs measured six months later (visit 2). However, no significant results were found in this analysis (data not shown).

### Interactions between different biomarkers that were associated with sexual exposure groups.

#### (1) Microbial abundances and plasma cytokine levels:

The differences in levels of inflammatory markers CRP, sCD14, and sCD163, previously established as significantly associated with the exposure variable (number of receptive anal intercourse partners), were not found to be significantly associated with the DA species among the sexual exposure groups, with two exceptions noted within the genera *Lachnospira* and *RFN20* (Supplementary Table 1). (2) *Microbial abundances and SCFA levels*: Changes in gut microbial abundances among the sexual exposure groups were found to significantly influence shifts in SCFAs. DA species *B. uniformis, Megasphaera spp*., and *Succinivibrio spp*. showed significant associations with changes in acetate and valerate, both of which were previously identified to have significant associations with the sexual exposure group. Also, marginal significance was observed for *Butyricimonas spp*. And *Paraprevotella spp*. (Table 2). (3) *Plasma cytokine and SCFA levels*: Lastly, we further probed the relationship between inflammatory cytokines (CRP, sCD14, and sCD163) and SCFAs (acetate and valerate). The results of this analysis revealed a marginally significant effect of acetate on the levels of these cytokines (Supplementary Table 2).

### Mediation analysis

The primary aim of this study was to assess whether biomarkers—namely inflammatory cytokines, gut microbiota, and SCFAs—mediate the relationship between an increase in the number of partners for receptive anal intercourse and HIV-1 seroconversion (refer to Extended Data Fig. 4a). We focused on specific biomarkers including cytokines sCD14 and sCD163, and DA species *A. muciniphila, B. caccae, B. fragilis, B. uniformis, Bacteroides spp., Butyricimonas spp., Dehalobacterium spp., Methanobrevibacter spp*., and *Odoribacter spp*. These biomarkers were selected since they were identified as having significant associations with both sexual exposure groups as well as the outcome variable, HIV-1 seroconversion status. Since our previous research^21^ did not identify any significant SCFAs associated with HIV-1 seroconversion, SCFAs were excluded from the mediation analysis.

Using the natural effect model, consisting of sexual exposure groups as the exposure variable, biomarkers (sCD14 and sCD163) as the mediators, and HIV-1 seroconversion status as the outcome variable, we discovered a significant natural direct effect (NDE) of the exposure on the outcome while controlling for biomarker levels. NDE quantifies the effect of the exposure on the outcome not through the mediator. Specifically, for a subject who had not used substance, increasing the exposure from Group 1 to another group (while maintaining sCD14 and sCD163 at the same level) significantly increased the odds of seroconversion. The odds ratios for Groups 2, 3, and 4 are exp(1.92) = 6.82, exp(2.56) = 12.94, and exp(3.55) = 34.81, respectively (Table 3). This increasing trend is highly significant with p-value < 0.001 (Table 3)^24^. In addition to NDE, we also investigated the natural indirect effect (NIE) of the exposure on the outcome that acts through the mediator. Unlike NDE, NIE represents the effect of the intervention on the outcome solely due to its effect on the mediator. The natural effect models indicated a significant NIE of sCD14 and sCD163 levels on HIV-1 seroconversion while controlling for the exposure among subjects who had the largest number of receptive anal intercourse partners (p = 0.02, Table 3). A trend analysis further strengthened this result with a significant increase (p = 0.007, Table 3) in NIE corresponding with biomarker levels for subjects that were exposed to a higher number of partners for receptive anal intercourse. Specifically, for a subject who had not used substance, shifting levels of sCD14 and sCD163 from those observed in Group 1 to those potentially seen in Group 4, while holding the exposure constant at any given group, increases the odds of seroconversion with an odds ratio of exp(0.33) = 1.39.

A similar natural effect model consisting of sexual exposure groups as the exposure variable, microbiota *A. Muciniphila, B. caccae, B. fragilis, B. uniformis, Bacteroides spp., Butyricimonas spp., Dehalobacterium spp., Methanobrevibacter spp*., and *Odoribacter spp*. as mediators, and HIV-1 seroconversion status as the outcome variable, we discovered a significant NDE of the exposure on the outcome while controlling the microbial abundances. For study participants who had not used substance, changing the exposure from Group 1 to any other group, while maintaining microbial abundance, increased the odds of seroconversion (odds ratios for Groups 2, 3, and 4 are exp(2.08) = 8.00, exp(2.61) = 13.60, and exp(3.58) = 35.87, respectively; Table 3). A trend analysis further strengthened this result with a significant increasing trend (p < 0.001, Table 3). Moreover, this model highlighted a significant NIE of microbial abundances on HIV-1 seroconversion, particularly among subjects with the largest number of receptive anal intercourse partners (p = 0.04, Table 3). Trend analysis further validated a notable increase in NIE associated with microbial abundance for subjects that were exposed to a greater number of receptive anal intercourse partners, with a p-value of 0.033 (Table 3). Specifically, transitioning microbial species abundances from those of Group 1 to those in Group 4, while holding exposure constant, increased seroconversion odds by exp(0.35) = 1.42.

When the cytokines and microbial species were taken together as mediators into the natural effect models, which is biologically reasonable to consider, the results of NDE for this combined model were very similar to NDE results when separate natural models were considered for cytokines and microbial species (Table 3). However, there was a substantial increase in the NIE estimates from the combined model consisting of cytokines and microbiomes as mediators (Table 3) when compared to the two sets of mediators, cytokines, and microbiomes, modeled separately. For example, the NIE estimates of cytokines and microbiomes in Group 4 relative to Group 1, was about 1.5 times the NIE estimates of either cytokines or microbiomes in Group 4 relative to Group 1, when modeled separately. Thus, the microbiome and the cytokines synergistically mediated the effects of sexual exposure on HIV seroconversion. Interestingly, sexual exposure groups had significant NDE on many microbial species and cytokines when taken individually (i.e., separate models for each of these variables) (Supplementary Table 3). However, individually, almost none of these variables mediate the effect of sexual activity on seroconversion (Supplementary Table 3). Taken together, the above results suggest that, rather than individually, collectively the biomarkers sCD14 and sCD163, and the microbial species *A. muciniphila, B. caccae, B. fragilis, B. uniformis, Bacteroides spp., Butyricimonas spp., Dehalobacterium spp., Methanobrevibacter spp*., and *Odoribacter spp*., mediate the effects of sexual behavior on HIV seroconversion.

## Discussion

In our previous study^21^, gut microbiome dysbiosis and increased levels of plasma inflammatory cytokines were observed in MACS MSM in the 6 months prior to their becoming infected with HIV-1, i.e., termed HIV-1 seroconverters). In this follow-up study, the sexual behaviors of the same study participants were closely examined to explore their association with the cytokine levels, SCFA levels, and gut microbiome composition. The results of our current study have revealed the intricate network of sexual behavior, immune response, and microbiota composition, which greatly impact one’s susceptibility to HIV-1 infection.

It was striking to see a reduction in the abundance of several species of the genera Bacteroides in the higher sexual exposure groups compared to men in the no exposure group (Group 1), i.e., those who did not have receptive anal intercourse (Fig. 4a). These species were also reduced in abundance in the SC group compared to NC (Fig. 4b). Many species of *Bacteroides* are commensal gut bacteria and play a prominent role in gut health^25^. For example, *B. fragilis* whose abundance was reduced in higher sexual exposure groups compared to men in the no exposure group (Group 1) and reduced in the SC group compared to NC, plays an important role in preventing the expansion of infected Th17 cells and protects from potential damage to mucosal barrier. Similarly, *B. uniformis*, which has reduced abundance in both high exposure groups as well as the SC group, is involved in the production of IL-10, TNF-alpha and in the protection against metabolic and immunological dysfunction^26^, and is thus considered to be of therapeutic importance^27^. Similar to various species of *Bacteroides*, there is a reduction in *Akkermansia muciniphila*, in the higher sexual exposure groups compared to men in the no exposure group (Group 1), i.e., those who did not engage in receptive anal intercourse (Fig. 4a) and this species was also reduced in abundance in SC group compared to NC (Fig. 4b). This species is known to be involved in numerous functions such as mucin degradation which is necessary to produce acetate and propionate, forming a substrate for other bacteria and host^28^. It is also involved in tissue repair of the intestinal mucosa and is thus important for the integrity of the intestinal mucosa^29^,is anti-inflammatory, involved in immunomodulation, and is involved in the regulation of body weight.

Interestingly, pairwise correlations among almost all these taxa decreased over the sexual exposure groups. Specifically, the estimated correlations in the highest sexual group (Group 4) were zero for many taxa (Extended Data Fig. 5). These findings, together with reduction in the abundance of *A. muciniphila, B. caccae, B. fragilis, B. uniformis, Bacteroides spp., Butyricimonas spp*., and *Odoribacter spp,* and an increase in the abundance proinflammatory species *Dehalobacterium spp*. and *Methanobrevibacter spp*., point to gut dysbiosis with an increase in the number of partners with whom a participant had receptive anal intercourse, leading to HIV seroconversion.

Our mediation analysis, employing natural effect models, illuminates the intricate interplay of sexual behavior, immune response, and gut microbiota in relation to HIV-1 seroconversion. We found that the number of receptive anal intercourse partners stands as a significant predictor of HIV-1 seroconversion, with remarkably large effect sizes for natural direct effects. Further, our study reveals significant natural indirect effects of two cytokines (sCD14 and sCD163) and multiple microbial species (*A. muciniphila, B. caccae, B. fragilis, B. uniformis, Bacteroides spp., Butyricimonas spp., Dehalobacterium spp., Methanobrevibacter spp*., and *Odoribacter spp*.) in men who engage in receptive anal intercourse with a large number of partners. This finding highlights the potential role of these biological markers as mediators in the relationship between high-risk sexual behaviors and HIV-1 seroconversion. Notably, these significant indirect effects were observed primarily among individuals engaging in more extreme risky sexual behaviors, such as having many receptive anal intercourse partners. Notably, although we considered a simplified causal pathway described in Extended Data Fig. 4a to serve as an exploratory mediation analysis for screening potential mediators, a more comprehensive causal pathway from risky sexual behavior to HIV-1 seroconversion described in Extended Data Fig. 4b needs to be considered. However, to perform such an analysis, more sophisticated methods are necessary which are beyond the scope of this study.

In our study, in addition to considering the number of receptive anal intercourse partners as the exposure, we also probed the status of other STIs and HBV as alternative exposures with respect to the outcome of HIV-1 seroconversion (data not shown). Our results revealed that STI status showed a marginally significant association with IL-6 (p = 0.05) and a significant association with SCFAs including acetate, butyrate, and valerate (p = 0.02, 0.005, and 0.03, respectively). Nevertheless, no significant differential abundance of microbial genera or species was observed in relation to STI status. On the other hand, HBV status (resolved vs. negative, positive group was excluded due to a small sample size) was marginally significantly associated with IL-6 (p = 0.05), significantly associated with CRP (p = 0.01), and significantly linked to differential abundances of the microbial genus *Alistipes* (p < 0.001) and two microbial species, *Parabacteroides stercorea* and *Alistipes putredinis* (both p < 0.001). Utilizing the natural effect models, we noted that while considering STI history, HIV-1 seroconversion, and biomarker mediators, a statistically significant natural direct effect was discerned. However, the natural indirect effect was not found to be significant, suggesting that STIs play a pivotal role in the pathway to HIV-1 seroconversion. This effect does not appear to be mediated by the levels of cytokines, gut microbiota, or SCFAs. Lastly, when implementing the natural effect models while considering HBV, HIV-1 seroconversion, and biomarker mediators, we identified a statistically significant, natural direct effect as well as a natural indirect effect for the microbial mediator *Alistipes*. This finding accentuates the crucial role of microbiome biomarkers on HIV-1 seroconversion.

These insights underscore the complexity of the factors contributing to HIV-1 seroconversion, demonstrating the interconnectedness of sexual behavior, immune response, and microbiota composition, especially among MSM participating in high-risk sexual behaviors^30^. This study impacts our understanding of the role of the microbiome and immune response in the context of HIV-1 transmission in the mid-1980s prior to the current public health emphasis on safer sex, including the use of condoms and pre-exposure prophylaxis (PrEP) with antiretroviral drugs^3^. Notably, our results are pertinent and timely regarding the contemporary risk for HIV-1 infection among MSM in that condomless sex has steadily increased in the last two decades^30,31^. Moreover, although PrEP is highly effective for preventing HIV-1 infection in MSM^32^, PrEP has been associated with aberrations in gut microbiota^33,34^. In conclusion, our results emphasize the myriad disruption of gut microbiota in PWH related to sexual activity and its significant contemporary consequences.

## Methods

### Study Participants

A total of 109 HIV-1 seroconverters (SC) and 156 HIV-1 seronegative controls (NC) from the MACS were included in this study. The MACS was a prospective cohort study of HIV-1 infection in MSM established in 1983 at 4 sites (Baltimore, Maryland/Washington, DC; Chicago, Illinois; Los Angeles, California; Pittsburgh, Pennsylvania)^7,35,36^, that combined with the Women’s Interagency HIV Study (WIHS) in 2019 to form the MACS-WIHS Combined Cohort Study (MWCCS)^37^. MACS participants were studied at semiannual clinic visits with standardized interviews, physical examinations, and phlebotomy for laboratory testing, with cryostorage of plasma and serum and viable peripheral blood mononuclear cells. The study was conducted with institutional review board approval from all participating institutions. Enrollment and clinical research of the MACS participants began April 1, 1984, with clinical research visits at 6-month intervals thereafter. During that early period of the AIDS pandemic, a substantial number of MACS participants acquired HIV-1 infection during study follow-up. Importantly, during this time HIV-1 serostatus could not be determined, as no HIV-1 specific diagnostic test was available. HIV-1 seroconversion was subsequently determined with the stored blood plasma samples by enzyme-linked immunosorbent assay and confirmed by Western blot^36^. The HIV-1 infection date was estimated as the midpoint between the last seronegative and first seropositive study visits.

### Collection of demographic, clinical, and behavior data of participants

During each study visit, study participants provided demographic, clinical, and behavior information. For the baseline visit, this information covered the past 2 years, whereas for all subsequent visits, it encompassed the period since the last visit (6 months). Participants were categorized into four ordered groups (Table 1) based on participant responses regarding the number of partners with whom a participant had receptive anal intercourse, the exposure variable of interest. Group 1 had no receptive anal intercourse partners (N = 63); Group 2 had one receptive anal intercourse partner (N = 57); Group 3 had 2 to 5 receptive anal intercourse partners (N = 86); and Group 4 had 6 or more receptive anal intercourse partners (N = 35). For simplicity of exposition, throughout this paper we shall use the phrase “sexual exposure groups” to mean the above four ordered groups.

### Stool and plasma samples

During this early phase of the HIV-1 pandemic in 1984–1985, MACS participants were instructed to provide stool, urine, semen, and oral wash samples at each clinic research visit; these have been preserved at −80C without additives or preservatives^36^. For this current study, the stool and plasma samples were obtained from specimen cryorepositories of the MACS. Stools were self-collected in 20 ml screw-capped glass vials at home and delivered to the clinic within one day by the participants. In the current study, we examined stool and plasma samples from the study visits flanking the estimated HIV-1 infection time point from 109 HIV-1 SCs, and from 156 HIV-1 uninfected, age and race matched, MSM participant controls collected during the same time period at the same MACS center. Plasma HIV-1 RNA loads were determined retrospectively by Roche quantitative RT-PCR with a detection limit of 300cp/ml or Roche Ultra-sensitive quantitative RT-PCR with detection limit of 40cp/ml. Similarly, plasma HBV core antibody (cAB), HBV surface antigen (sAg), and HCV antibody (Ab) were also tested retrospectively by commercially available ELISA tests. The paired samples spanned approximately 6 months, with the 3-month midpoint designated as the date of HIV-1 seroconversion. Of the 109 HIV-1 SCs, 32 developed AIDS^38^ within 5 years after seroconversion, 31 developed AIDS within 5–10 years, and 46 were AIDS-free for more than 10 years after seroconversion, all without antiretroviral drug therapy (ART).

### Profiling microbial populations by sequencing of the variable region of the 16S rRNA gene

Fecal DNA was extracted from the stool samples using the PowerSoil DNA Extraction Kit (MO BIO Laboratories, Carlsbad, CA, USA). The V4 variable region in the 16S rRNA gene was PCR-amplified with the universal primers: 515F 5’-(GTG CCA GCM GCC GCG GTA A)-3’ and 806R 5’-(GGA CTA CHV GGG TWT CTA AT)-3’^39^. DNA concentrations were measured using Qubit 4 Fluorometer (Thermo Fisher Scientific, Waltham, MA, USA). Amplicons were cleaned, pooled and sequenced on an Illumina MiSeq platform according to the manufacturer’s specifications to generate paired-end reads. The datasets generated and/or analyzed during the current study are available in the GitHub repository^40^.

### 16S rRNA gene sequence analysis

The resulting 16S rRNA gene sequence data were processed using QIIME2 (version 2019.10.0). The raw sequence data were first demultiplexed and then denoised to remove noisy reads and dereplicate sequence, and clustered into amplicon sequence variants (ASVs) using the DADA2 algorithm^41^. The observed counts of ASVs were organized into a large matrix referred to as the feature table, where columns represent samples and rows represent ASVs. No ASV was removed based on its observed abundance. The taxonomic composition of bacterial communities was investigated by classifying sequences to the latest reference database^42^ using a Naive Bayes classifier.

### Fecal SCFA measurement

To determine the concentration of SCFAs in the stool samples, 50–100mg of fecal matter were aliquoted from every stool sample and sent to the University of Pittsburgh Health Sciences Metabolomics and Lipidomics Core in the Department of Pharmacology & Chemical Biology. The fecal acetate, butyrate, propionate and valerate levels were measured by stable isotope dilution liquid chromatography mass spectrometry^43^.

### Measurement of plasma inflammatory cytokines

The inflammatory cytokines soluble CD14 (sCD14), soluble scavenger receptor CD163 (sCD163), C-reactive protein (CRP), interferon γ-induced protein 10 (IP-10) and lipopolysaccharide binding protein (LBP), were measured with the Luminex xMAP platform (Luminex, Northbrook, IL, USA), according to the manufacturer’s instructions. The data were collected and analyzed using a BioPlex 200 apparatus and BioPlex Manager Software (Bio-Rad, Hercules, CA, USA). In addition, the inflammatory cytokine interleukin 6 (IL-6) was measured in the plasma samples by ELISA using a commercially available ELISA kit (R&D, Minneapolis, MN, USA) following the manufacturer’s instructions.

### Statistical methods

Unless otherwise noted, all analyses presented in this study were performed using data from the MACS clinic baseline visit 1. A significance threshold was set at α=0.05. Multiple testing corrections were not applied to the p-values unless explicitly specified. Also, unless stated otherwise, all analyses in this article were carried out using the default settings in the respective software packages.

#### Marginal association between demographic and clinical features and exposure groups and the outcome

Ordinal logistic regression models were applied to investigate the marginal associations between the ordered sexual exposure groups and individual demographic and clinical features. This analysis utilized the “polr” function from the “MASS” package in R^44^. Likewise, logistic regression models were employed to explore the marginal associations between the outcome (SC vs. NC) and individual demographic and clinical features. This analysis was conducted using the “glm” function from the “stats” package in R.

#### Analysis of gut microbiota diversity, differential abundance (DA), and correlations

Alpha (within-sample) and beta (between-sample) diversity indices were calculated utilizing the R “microbiome” package^45^ applied to rarefied data at the species level. Specifically, species were subsampled without replacement, anchored at 90% of the minimum library size. For the alpha diversity, we considered both Shannon diversity index as well as species richness. Adjusting for bacterial antibiotics usage, trends over the ordered sexual exposure groups, and pairwise comparisons among the sexual exposure groups, all while, were performed using the “CLME” R package^46^. For the beta diversity, we used Bray-Curtis dissimilarity measure and the statistical significance was determined through pairwise Permutational Multivariate Analysis of Variance^47^. Bacterial species displaying monotonically increasing or decreasing trends over the four sexual exposure groups were identified by the Analysis of Compositions of Microbiomes with Bias Correction 2^48^ without applying a sensitivity score filter. Bacterial antibiotics usage was adjusted as a covariate. The p-values associated with these trends were estimated based on 1000 bootstrap samples. Additionally, ANCOM-BC2 without a sensitivity score filter was employed to identify DA species between SC and NC groups. Pairwise Pearson correlations between species were estimated using Sparse Estimation of Correlations among Microbiomes (SECOM)^49^. Species yielding raw p-values < 0.05 were shown in the corresponding ANCOM-BC2 results. Those that remained significant after adjustment for multiple comparisons using the Benjamini–Yekutieli procedure^50^ are highlighted in the figure.

#### Analysis of SCFA and inflammatory cytokine difference between groups

The levels of SCFA and inflammatory cytokines were evaluated across sexual exposure groups. Trend analysis over the four sexual exposure groups were performed using CLME while adjusting for bacterial antibiotics usage.

#### Interactions between different biomarkers

To investigate the multivariate relationships among cytokines, SCFAs, and the microbial species that were identified as associated with sexual exposure groups, the multivariate analysis of covariance (MANCOVA) using Pillai-Bartlett trace statistic was performed^51^. The sample and taxon specific bias-corrected log microbial abundances obtained from the ANCOM-BC2 model were utilized in this investigation.

#### Mediation analyses

To understand whether changes in biomarker levels (microbiome, SCFA, and inflammatory cytokines) mediate the effect of sexual exposure groups on the outcome (HIV-1 seroconversion), we employed Natural Effect Models^52–55^. We focused on the potential mediating effects of biomarkers (cytokines, the gut microbiome, and SCFAs) that were significantly associated with the sexual exposure group as well as the outcome variable, namely, HIV-1 seroconversion status. Based on the results of univariable analyses involving demographic, clinical, and behavior features, sexual exposure groups, and the seroconversion status outcome (Fig. 2), we identified substance use as the confounding variable for inclusion in the mediation analyses. It is crucial to highlight that the analysis of high-dimensional and compositional mediators, such as microbiome compositions, is an active research area and presents significant challenges. While several methodologies have been proposed, including MarZIC^56^, CCMM^57^, and SparseMCMM^58^, computational software is not yet available. As a result, in this study, we utilized Natural Effect Models, which has established and accessible software. We performed the above mediation analysis using the R package “medflex”^59^. Imputations for counterfactuals were performed using the function “neImpute” with the setting “robust” for “se” to ensure robust standard errors for the parameter estimates.

## Extended Data

**Extended Data Fig. 1:** Information on this HIV study cohort and study participants. At visit one, demographic information (age, gender, race) was collected; At visits one to four, personal information, including sexual activity, alcohol usage, substance use, was collected. At visit one through visit three, blood, oral wash, urine, and stool samples were collected from study participants. MACS: Multicenter AIDS Cohort Study, WIHS: Women’s Interagency HIV Study, MWCCS: MACS-WIHS Combined Cohort Study.

**Extended Data Fig. 2:** Bar plots showcase monotonically increasing or decreasing trends between exposure groups and microbial alpha diversities at the species level. **(a)** species richness with an increasing trend, **(b)** Shannon diversity index with an increasing trend, **(c)** species richness with a decreasing trend, and **(d)** Shannon diversity index with a decreasing trend. The X-axis defines the exposure groups, spanning from Group 1 (G1) to Group 4 (G4). The Y-axis indicates the alpha diversity’s effect size, either richness or Shannon index, as gauged by the constrained linear mixed effects (CLME) model. Error bars on each bar represent the 95% confidence interval (CI). Pairwise p-values (one-sided), contrasting the exposure groups, are illustrated above the brackets encompassing the corresponding bars. The plot includes the overall p-value for the monotonic trend evaluation. Notably, the analyses revealed no significant associations with the alpha diversity metrics.

**Extended Data Fig. 3:** PCoA plot of the microbial beta diversity (Bray-Curtis dissimilarity) at the species level. Each point corresponds to an individual subject, color-coded by their exposure groups. Ellipses cover 80\% of the data distribution. Adjacent tables detail contrasts, p-values ascertained through pairwise Permutational Multivariate Analysis of Variance (PERMANOVA), and adjusted p-values (q-values) that are rectified utilizing the Benjamini-Hochberg (BH) procedure.

**Extended Data Fig. 4:** Directed Acyclic Graph (DAG) depicting the mediation analyses. **(a)** Represents the working model employed in the current study, while **(b)** illustrates a theoretically robust model that we proposed. However, the latter was not implemented in this paper due to constraints in the statistical methodology.

**Extended Data Fig. 5:** SECOM results of pairwise Pearson correlation coefficients between differentially abundant species. The X-axis denotes the exposure groups, ranging from Group 1 (G1) to Group 4 (G4). The Y-axis conveys the Pearson correlation coefficients determined by Sparse Estimation of Correlations among Microbiomes (SECOM). Precise correlation values are displayed above each data point. The plot exclusively showcases pairwise correlations with at least one non-zero value across the groups.

## Figures and Tables

**Table 1.** Summary of demographic, clinical, and behavior features

		N = 243
Age (mean (sd))		40.70 (16.11)
Race (n (%))		
	White	230 (95)
	Black	8 (3)
	Others	4 (2)
	Missing	1 (0)
Education (n (%))		
	Postgrad	95 (39)
	Under grad	58 (24)
	No degree	88 (36)
	Missing	2 (1)
Smoking Status (n (%))		
	Never	97 (40)
	Former	42 (17)
	Current	103 (42)
	Missing	1 (0)
Drinking Status (n (%))		
	Low	103 (42)
	Heavy	136 (56)
	Missing	4 (2)
Antibiotics Usage (n (%))		
	No	128 (53)
	Yes	115 (47)
STD (n (%))		
	No	203 (84)
	Yes	39 (16)
	Missing	1 (0)
Substance Use (n (%))		
	No	45 (19)
	Yes	197 (81)
	Missing	1 (0)
HBV		
	Negative	96 (40)
	Resolved	133 (55)
	Positive	8 (3)
	Missing	6 (2)
HCV		
	Negative	231 (95)
	Positive	6 (2.5)
	Missing	6 (2.5)
Sexual Exposure Group		
	G1	63 (26)
	G2	57 (23)
	G3	86 (35)
	G4	35 (14)
	Missing	2 (1)

**Table 2.** MANCOVA analysis comparing bias-corrected abundances of DA species with significant SCFA levels.

Species	F statistic	Num. d.f.	Den. d.f.	P-value
*A.mucimphila*	0.46	2	196	0.63
*A*.*onderdonkii*	0.2	2	196	0.82
*Anaerovibrio.spp*.	0.69	2	196	0.50
*B.adolescentis*	0.14	2	196	0.87
*B.caccae*	0.95	2	196	0.39
*B.fragilis*	2	2	196	0.13
*B.unifonnis*	6.3	2	196	0.0022**
*Bacteroides. spp*.	1.2	2	196	0.30
*Bifidobacterium, spp*.	0.17	2	196	0.85
*Biityricimonas. spp*.	2.5	2	196	0.088
*C.celatum*	0.89	2	196	0.41
*Dehalobactenum.spp*.	1.5	2	196	0.22
*Lachnobacierium. spp*.	0.17	2	196	0.85
*Lachnospira. spp*.	0.34	2	196	0.71
*Megasphaera. spp*.	3.6	2	196	0.029*
*Methanobrevibacter. spp*.	1.7	2	196	0.18
*Odoribacter.spp*.	0.17	2	196	0.84
*Paraprevotella.spp*.	2.4	2	196	0.094
*RFN20.spp*.	1.7	2	196	0.19
*Succinivibrio.spp*.	3.4	2	196	0.036*

Abbreviations: Num. d.f. = numerator degrees of freedom; Den. d.f. = denominator degrees of freedom. Significance levels are denoted as follows:

* for p<0.05,

** for p<0.01, and

*** for p<0.001.

**Table 3.** Results of the natural effect models consisting of sexual exposure groups as the exposure variable, biomarkers (cytokines, microbial species, or their combined effects) as the mediators, and HIV-1 seroconversion status as the outcome variable. LOR: log odds ratio (natural log base). Trend test p-values were derived using the methodology detailed in Peddada et al.

	Natural Direct Effect (NDE)				
Mediator	Comparison	LOR	SE	P-Value	Trend Test P-Value
	G2 – G1	1.92	0.64	0.003	
sCD14, sCDl63	G3 – G1	2.56	0.61	0.001	<0.001
	G4 – G1	3.55	0.7	0.001	
*A.muciniphila*, *B. caccae,*					
*B.fragilis*, *B.uniformis*, *Bacteroides spp*.,	G2 – G1	2.08	0.68	0.002	<0.001
*Butyricimonas spp., Dehalobacterium spp*.,	G3 – G1	2.61	0.64	0.001	
*Methanobre.vibac.ter spp., Odoribacter spp*.	G4 – G1	3.58	0.71	0.001	
sCD14, sCD163,					
*A.muciniphila, B. caccae,*					
*B.fragilis, B.uniformis*,	G2 – G1	1.81	0.64	0.005	<0.001
*Bacteroides spp*.,					
*Butyricimonas spp*.,	G3 – G1	2.38	0.61	0.001	
*Dehalobacterium spp*.,					
*MelhariobrevibacLer spp*.,	G4 – G1	3.16	0.68	0.001	
*Odoribacter spp*.					
	Natural Indirect Effect (NIE)				
Mediator	Comparison	Estimate	SE	P-Value	Trend Test P-Value
	G2 – G1	0.11	0.1	0.284	
sCD14, sCD163	G3 – G1	0.12	0.09	0.168	0.007
	G4 – G1	0.33	0.14	0.023	
*A.muciniphila, B. caccae*,					
*B.fragilis, B. uniformis,*					
*Bacteroides spp*.,	G2 – G1	0.02	0.13	0.879	
*Butyricimonas spp*.,					0.033
*Dehalobacterium spp*.,	G3 – G1	0.15	0.13	0.264	
*Methanobrevibacter spp*.,					
*Odoribacter spp*.	G4 – G1	0.35	0.17	0.045	
sCD14, sCD163,					
*A.muciniphila, B. caccae*,					
*B.fragilis, B.uniformis,*	G2 – G1	0.2	0.17	0.241	0.001
*Bacteroides spp*.,					
*Butyricimonas spp*.,	G3 – G1	0.29	0.17	0.087	
*Dehalobacterium spp*.,					
*Methanobrevibacter spp*.,	G4 – G1	0.74	0.24	0.002	
*Odoribacter spp*.					

## Data Availability

The processed datasets utilized in the present study have been made accessible through our GitHub repository^40^. The raw data, including the microbiome sequencing data, are not publicly available but may be obtained by contacting Dr. Chen Yue (cheny@pitt.edu).
